# Peptide Cotransmitters as Dynamic, Intrinsic Modulators of Network Activity

**DOI:** 10.3389/fncir.2018.00078

**Published:** 2018-10-02

**Authors:** Elizabeth C. Cropper, Jian Jing, Ferdinand S. Vilim, Klaudiusz R. Weiss

**Affiliations:** ^1^Department of Neuroscience and Friedman Brain Institute, Icahn School of Medicine at Mount Sinai, New York, NY, United States; ^2^State Key Laboratory of Pharmaceutical Biotechnology, Advanced Institute for Life Sciences, School of Life Sciences, Nanjing University, Nanjing, China

**Keywords:** neuropeptide, cotransmitter, neuromodulation, invertebrate, autonomic nervous system

## Abstract

Neurons can contain both neuropeptides and “classic” small molecule transmitters. Much progress has been made in studies designed to determine the functional significance of this arrangement in experiments conducted in invertebrates and in the vertebrate autonomic nervous system. In this review article, we describe some of this research. In particular, we review early studies that related peptide release to physiological firing patterns of neurons. Additionally, we discuss more recent experiments informed by this early work that have sought to determine the functional significance of peptide cotransmission in the situation where peptides are released from neurons that are part of (i.e., are intrinsic to) a behavior generating circuit in the CNS. In this situation, peptide release will presumably be tightly coupled to the manner in which a network is activated. For example, data obtained in early studies suggest that peptide release will be potentiated when behavior is executed rapidly and intervals between periods of neural activity are relatively short. Further, early studies demonstrated that when neural activity is maintained, there are progressive changes (e.g., increases) in the amount of peptide that is released (even in the absence of a change in neural activity). This suggests that intrinsic peptidergic modulators in the CNS are likely to exert effects that are manifested dynamically in an activity-dependent manner. This type of modulation is likely to differ markedly from the modulation that occurs when a peptide hormone is present at a relatively fixed concentration in the blood.

## Introduction

For more than 40 years, it has been apparent that neurons can contain both neuropeptides and “classic” small molecule transmitters. Much progress has been made in studies designed to determine the functional significance of this arrangement in experiments conducted in invertebrates and in the vertebrate autonomic nervous system. Below we describe some of these key results. We begin by discussing early experiments that studied cotransmission in preparations in which it was possible to directly monitor peptide release. These data provided (still valid) insights into the dynamics and pattern dependance of peptide release that could not be obtained in less experimentally advantageous systems. Later sections of this review article then describe how these insights have informed more recent research that has sought to determine the physiological role of peptide cotransmitters that are intrinsic to a behavior-generating network.

## Peptide Release

### Does Peptide Release Occur During Normal Behavior?

It has long been apparent that neuropeptides can coexist with small molecule neurotransmitters. For example, Hökfelt and coworkers reported somatostatin-like immunoreactivity in noradrenergic neurons in principle ganglion cells of sympathetic ganglia in 1977 (Hökfelt et al., 1977). The demonstration of coexistence then led to the obvious question, do “co-existing” peptides function as neurotransmitters? (an alternative possibility would be that they simply act as trophic factors).

In the early 1980s the cotransmission question was addressed by Jan and Jan (1982) in experiments in the sympathetic nervous system of the bullfrog. There is general agreement that certain criteria have to be met for a substance to be classified as a neurotransmitter. Although there is some disagreement as to how many of these criteria there are, Jan and Jan (1982) were able to convincingly satisfy those that are most commonly considered crucial. For example, they demonstrated that the peptide they studied (LH-RH) is present in presynaptic terminals, and is released in a calcium dependent manner, Further, a late, slow EPSP was mimicked by application of exogenous LH-RH, and blocked by LH-RH antagonists (for a detailed discussion of this work see Nusbaum, 2017).

A further question that was subsequently raised was, under what circumstances does peptide release occur? Early experiments in the sympathetic nervous system of the pig used a radioimmunoassay (RIA) to quantify NPY-like immunoreactivity in the perfusate following low frequency (i.e., 2 Hz) nerve stimulation as compared to release induced by intermittent stimulation at a high frequency (20 Hz; Lundberg et al., 1986). Release was greater at the higher frequency. Data such as these led to the hypothesis that peptide release only occurs if neurons fire at high/excessive frequencies that are observed under pathological conditions (Hökfelt, 1991). This line of thinking led to the prediction that peptides would only be important for mediating responses to injury or stress (Hökfelt, 1991).

Subsequent invertebrate research clearly established that this is not the case. Many of these early studies were conducted in neuromuscular systems (O’Shea and Schaffer, 1985). An initial goal of this work was to verify peptide release by making direct biochemical measurements. Peptide release was induced either by raising the potassium concentration in the saline, or by stimulating motor neurons at relatively high frequencies. For example, Adams and O’Shea (1983) demonstrated proctolin release from a slow skeletal motoneuron (Ds) in the cockroach with stimulation at 50 Hz. Other lower frequencies were not tested when release was directly monitored, presumably because the method used to detect released peptides was not very sensitive. However, in other experiments in this study, peptide release was monitored indirectly, i.e., by monitoring a physiological response clearly not mediated by the release of the primary neurotransmitter (glutamate). Thus, Adams and O’Shea (1983) also demonstrated that when a burst of action potentials is triggered in Ds, a delayed slow increase in muscle tension is observed that is not associated with excitatory junctional potentials (EJPs). This delayed response was observed when Ds was stimulated at a frequency that was not specified but was clearly way below 50 Hz.

In another early invertebrate study, proctolin release was monitored in a neuromuscular preparation of the crayfish using a sensitive and quantifiable bioassay, i.e., samples were applied to a subset of muscle fibers from the main extensor muscle of the locust leg and changes in contraction frequency were noted (O’Shea and Bishop, 1982). In this situation, it was possible to detect release when motor neurons were stimulated at a fairly low frequency (e.g., 10 Hz; Bishop et al., 1987).

Other experiments were conducted in a molluscan (*Aplysia*) preparation that consists of a muscle utilized in feeding, the accessory radula closer (ARC) and its two cholinergic motor neurons (B15 and B16; Cohen et al., 1978). Initially, peptide release in this system was monitored indirectly. For example, investigators measured cAMP levels in the ARC muscle (Whim and Lloyd, 1989; Cropper et al., 1990b). In later studies, however, a sensitive RIA was developed that permitted direct detection of released material (Vilim et al., 1996a).

**Figure 1:**
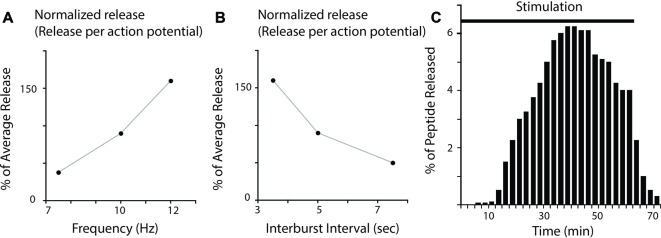
Peptide release in *Aplysia* neuromuscular preparations. **(A)** Effect of firing frequency on peptide release. Release was measured at three different firing frequencies in experiments in which the burst duration and interburst interval were kept constant. Plotted are results corrected to give the release per action potential. Note that there is more release when firing frequency increases (results are replots of data from Vilim et al. (1996a); error bars were omitted for clarity). **(B)** Effect of interburst interval on peptide release. Release was measured at three different interburst intervals in experiments in which the burst duration and firing frequency were kept constant. Plotted are results corrected to give the release per action potential. Note that increases in interburst interval decrease peptide release (results are replots of data from Vilim et al. (1996a); error bars were omitted for clarity). **(C)** Peptide release in response to intracellular stimulation of an accessory radula closer (ARC) motor neuron (i.e., stimulation at 12 Hz for 3.5 s every 7 s). The bar indicates the period of neural stimulation. Samples of muscle perfusate were collected every 2.5 min and peptide content was determined using a radioimmunoassay (RIA). Peptide release is expressed as percentage of total release in each experiment. Note that peptide release facilitated greatly and then declined until stimulation ceased (results are replots of data from Karhunen et al. (2001); error bars were omitted for clarity).

Research in the ARC neuromuscular system was unusual in its emphasis on mimicking naturally occurring patterns of motor neuron activity. Thus, extra junctional currents (EJCs) induced by B15 and B16 were recorded from the ARC muscle during normal feeding behavior in intact animals (Cropper et al., 1990a). Physiologically relevant patterns of neural activity were then simulated in subsequent release experiments that confirmed that the amount of peptide released depends on firing frequency in the ARC neuromuscular system (Vilim et al., 1996a, 2000) as it does in bullfrog sympathetic ganglia (e.g., Lundberg et al., 1986, 1989; Peng and Horn, 1991; Figure 1A). Importantly, however, release did occur at the low end of the physiological range (which is 7.5 Hz for B15 and 10 Hz for B16 (Cropper et al., 1990a; Vilim et al., 1996a,b, 2000). These data provide direct evidence for the release of peptides at physiologically relevant levels of activity, and obviously contradict the idea that peptide cotransmitters solely mediate responses to stress.

### Plasticity in Peptide Cotransmitter Release

It has long been recognized that neuropeptides are generally packaged in dense core vesicles whereas small clear vesicles generally contain low molecular weight neurotransmitters. In a number of neurons, data suggest that exocytosis from the two types of vesicles occurs in different regions in the presynaptic terminal. In some cases, release from the small clear vesicles occurs in the active zone and release from peptidergic large dense core vesicles appears to occur elsewhere (e.g., Zhu et al., 1986; Vilim et al., 1996b; Lysakowski et al., 1999; Karhunen et al., 2001). Further, release from the two types of vesicles is often differentially sensitive to increases in the intracellular calcium concentration. Release from peptidergic large dense core vesicles occurs at lower [Ca^2+^]_i_ (Verhage et al., 1991; Peng and Zucker, 1993; Ohnuma et al., 2001). These data suggest that the patterning of neural activity could impact peptide cotransmitter release in a manner that differs from its impact on the release of a low molecular weight transmitter.

As described above, a number of investigators have demonstrated that more peptide is released when neurons fire at higher frequencies (Lundberg et al., 1986, 1989; Peng and Horn, 1991; Vilim et al., 1996a, 2000). Obviously as firing frequency increases, there is an increase in the number of action potentials triggered in a given period of time. One method that has been used to correct for this is to calculate the amount of peptide released per action potential. Even with this correction more release at higher firing frequencies has been demonstrated in the ARC neuromuscular system (if neurons are stimulated within a physiologically relevant range; Figure 1A; Vilim et al., 1996a, 2000).

An additional question that has been addressed is, are periods of rest necessary to maintain peptide cotransmitter release? That this could be the case had been suggested by experiments that studied peptide hormone release from the hypothalamus (Cazalis et al., 1985). Investigators working in the bullfrog sympathetic ganglia demonstrated that rest periods are not essential, i.e., LHRH-induced slow currents were recorded from postsynaptic neurons when presynaptic neurons were stimulated continuously (Peng and Horn, 1991).

Subsequently, research conducted in the ARC neuromuscular system elaborated on these findings (Vilim et al., 1996a, 2000). With excessive stimulation, depletion of peptide cotransmitters obviously occurs. Experiments were, however designed so that all parameters chosen were behaviorally relevant. For example, motor neurons were not fired at frequencies higher than those observed during normal behavior and burst durations and interburst intervals were all within physiological limits. Under these conditions, periods of rest were actually detrimental, i.e., there was a decrease in the amount of peptide released per action potential as the interburst interval was increased (Figure 1B). This result suggests that effects of modulatory neuropeptides will be manifested in a manner that is at least to some extent determined by how a behavior is executed. Namely, if it is executed rapidly, effects of peptides will be more pronounced.

Additionally, ARC investigators characterized the dynamics of peptide release when neural activity was maintained for a relatively long period of time (e.g., ~ an hour) with no change in either the motor neuron firing frequency or bursting pattern (Figure 1C; Vilim et al., 1996b, 2000; Brezina et al., 2000; Karhunen et al., 2001). In some of these experiments, motor neurons were stimulated at the high end of the physiological range, and pauses between bursts of activity were on the short side. Nevertheless, initially relatively little peptide was released. Over time however release facilitated and reached a peak (Figure 1C). Thereafter, it declined. These data suggest that even when behavior is constant, modulatory effects of peptide cotransmitters will be dynamically manifested. When a behavior is initiated, it may not be greatly impacted by peptide release. However, as it is repeated, peptidergic effects may become more pronounced (up to a point).

Taken together, these results indicate that neuropeptides are released during normal behavior. The amount of peptide released per action potential can vary greatly and be altered by the firing pattern of the neuron. Consequently, peptide release is likely to be determined by how behavior is executed (e.g., quickly or slowly). Additionally, even when patterns of neural activity do not change, peptide release may occur dynamically, e.g., effects of modulatory peptides may become more pronounced as a behavior progresses. Below we discuss potential functional consequences of these forms of plasticity in a specific situation—in the situation where peptide cotransmitters are intrinsic to a behavior generating circuit.

## Peptide Cotransmitters can be Intrinsic to a Behavior Generating Circuit

Modulatory neuropeptides are not always released as cotransmitters. In some well-characterized situations, they are released as hormones into the blood. For example, this is the case for some of the peptides that configure activity in the well-studied crustacean stomatogastric ganglion (STG; e.g., Christie et al., 1995; Marder and Bucher, 2007). This ganglion contains neurons utilized during feeding (e.g., chewing) and is located in an artery that is exposed to any substance present in the hemolymph. To give another well-characterized example, it is also the case for peptides such as eclosion hormone (EH) and ecdysis trigger hormone (ETH) that control ecdysis in insects (for review see, Taghert and Nitabach, 2012). It has therefore been suggested that neuropeptides typically act from outside motor networks to modulate output (Taghert and Nitabach, 2012).

Whether or not this is true depends on what is meant by “motor network.” For example, not all modulatory input to the STG is blood borne. Peptides are also present in projection neurons that innervate this ganglion and drive activity. For example, the GABA containing modulatory commissural neuron 1 (MCN1) also contains proctolin and *C. borealis* tachykinin-related peptide 1a (CabTRP1a; Blitz et al., 1999). Experiments in intact animals have demonstrated that MCN1 is involved in the processing of exteroceptive sensory input and influences motor activity under behaviorally relevant conditions (Hedrich et al., 2011). Chemosensory stimulation of the antennae of the crab increases the MCN1 firing frequency. It also triggers a gastric mill rhythm under normal conditions, but not if the MCN1 is lesioned. Thus, MCN1 may not be part of the “motor” gastric mill network. It is however clearly part of the behavior generating circuitry as a whole.

In a similar vein, a number of cerebral buccal interneurons (CBIs) in the mollusc *Aplysia* are peptidergic (e.g., Phares and Lloyd, 1996; Morgan et al., 2000; Vilim et al., 2001; Koh et al., 2003; Jing et al., 2010). These cells are also projection neurons and at least some of these neurons are activated by food under physiological relevant conditions and trigger motor activity (Rosen et al., 1991; Jing and Weiss, 2005; Wu et al., 2014). For example, one cholinergic neuron (CBI-2) is a command-like neuron that can drive ingestive responses (Rosen et al., 1991; Jing and Weiss, 2005).

Lastly, peptide cotransmitters have been localized to motor neurons and sensory neurons in a number of species. Peptide-containing motor and sensory neurons are not always part of the pattern generating circuit. However, motor neurons are obviously essential for the execution of behavior and sensory neurons often trigger it. In summary, although there are a number of well-characterized examples where peptides act from outside a behavior-generating network (e.g., function as hormones), there are also clear examples of situations in which they are intrinsic to the circuit that generates a particular behavior.

## Intrinsic vs. Extrinsic Modulator Release

A distinction between extrinsic and intrinsic modulation was originally made in the feeding system of *Aplysia* (Cropper et al., 1990b). The comparison there was between modulatory effects mediated by peptide cotransmitters in the ARC motor neurons, and modulatory input from the serotonergic metacerebral cells (MCCs). Peptide cotransmitters are obviously intrinsic to the behavior generating circuit. The MCCs were referred to as extrinsic because they are not part of the behavior generating circuitry *per se*. MCC activity does not induce a muscle contraction (Weiss et al., 1978). Further, feeding behavior is observed when the MCCs are lesioned (Rosen et al., 1983, 1989).

One difference between the two types of modulatory input arises from the fact that the release of an intrinsic modulator is likely to be tightly coupled to the manner in which the behavior is executed. This is particularly likely to be true for modulators such as peptide cotransmitters that are released in a pattern dependent manner. For example, the data reviewed above suggest that if behavior is executed rapidly, peptide release is likely to “automatically” increase. Further, if a behavior is maintained rather than terminated quickly, peptide release will progressively increase (at least for a while).

In contrast, it is not likely that such tight coupling will be observed with the release of an extrinsic modulator. In the ARC example cited above, the MCC firing frequency is at least in part determined by input that it receives from a sensory neuron that does not drive feeding motor programs (Chiel et al., 1986; Weiss et al., 1986a,b; Jing et al., 2008). Consequently, the MCCs are activated during feeding, but the MCC firing frequency is not tightly linked to variations in the activity of the behavior mediating feeding circuitry itself (Kupfermann and Weiss, 1982).

## Release of “Intrinsic” Peptide Cotransmitters From Projection Neurons

A further question is, what is the functional significance of intrinsic peptidergic neuromodulation? Obviously, the answer to this question will depend on the type of neuron that contains the peptide cotransmitter. For example, peptide cotransmitters released by motor neurons and sensory neurons are apt to exert relatively constrained effects. For example, peptides released by motor neurons are likely to modify the neuromuscular transform of one particular neuromuscular unit. In contrast, peptides released by projection neurons can exert effects that are widespread. For example, in the feeding circuit of *Aplysia* the peptides released by CBI-2 (feeding circuit activating peptide (FCAP) and cerebral peptide 2 (CP-2)) modify activity in a number of circuit elements (Morgan et al., 2000; Koh et al., 2003; Koh and Weiss, 2005, 2007; Friedman and Weiss, 2010). A more specific question is, how does peptide release from a projection neuron differ from a situation in which a modulatory peptide is released as a hormone?

**Figure 2:**
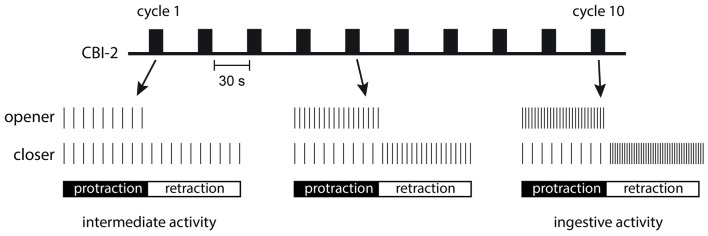
Repetition priming is observed when cycles of activity are triggered with an inter-burst interval of 30 s as is indicated in the schematic at the top of the figure. The first cycle that is induced is referred to as having intermediate characteristics. Motor neurons fire at low frequencies and radula opener and closer motor neurons are coactive (as is schematically illustrated in the bottom two rows on the left). With repeated motor program induction, activity becomes ingestive. Radula opener motor neurons are more active during the radula protraction phase of the motor program, and radula closer motor neurons are primarily active during radula retraction (as is schematically illustrated in the bottom two rows on the right).

In the *Aplysia* feeding circuit, FCAP and CP-2 act together to configure motor activity and make motor programs ingestive. Interestingly this occurs dynamically. Thus, when a single cycle of motor activity is triggered by CBI-2, motor neurons fire at relatively low frequencies and phase relationships are not very well defined (Figure 2; Proekt et al., 2004, 2007; Friedman and Weiss, 2010; Dacks et al., 2012). This type of motor activity is referred to as having intermediate characteristics. However, if CBI-2 is repeatedly stimulated with a relatively short interburst interval, program definition occurs (Figure 2; Proekt et al., 2004, 2007; Friedman et al., 2009; Friedman and Weiss, 2010; Dacks et al., 2012). The configuration of motor activity happens progressively with cycles of activity becoming more and more ingestive as they are repeatedly evoked. In other words, a form of repetition priming is observed, i.e., performance improves as behavior is repeated.

It is possible that postsynaptic events are partially responsible for the repetition priming that is observed in the feeding network. The CBI-2 peptides exert second messenger-mediated effects that may summate and become progressively larger when the interburst interval is short (Cropper et al., 2014). It is, however, very likely that plasticity in peptide release at least influences this process. Since effects of modulatory peptides are dose-dependent, we propose that progressive increases in the amount of peptide released are likely to impact function. This sort of progressive, activity-dependent change in the amount of peptide released is generally not observed when a peptide is released as a hormone. In conclusion, when peptides are released as cotransmitters from within a behavior mediating circuit, activity-dependent, dynamic effects may be observed that are not typical of peptide hormones. These effects may be important for the induction of phenomena such as repetition priming.

## Summary

Research conducted in invertebrates and in the vertebrate autonomic nervous system has played an important role in establishing that modulatory neuropeptides can function as cotransmitters and influence the generation of normal behaviors such as feeding and digestion. For example, peptides configure and reconfigure network activity and promote multitasking. Further, studies discussed in this review article have demonstrated that peptide release can be pattern and time dependent when neurons fire in physiologically relevant patterns. When peptide cotransmitters are intrinsic to a behavior generating circuit, this necessarily links peptidergic modulation to the manner in which a behavior is executed. For example, peptide release is more likely to occur when behavior occurs quickly. Further, when behavior is maintained, there can be progressive time-dependent increases in peptide cotransmitter release.

## Author Contributions

EC wrote the article with editorial suggestions from JJ, FV and KW.

## Conflict of Interest Statement

The authors declare that the research was conducted in the absence of any commercial or financial relationships that could be construed as a potential conflict of interest.
